# Exploring metabolic dysfunction in chronic kidney disease

**DOI:** 10.1186/1743-7075-9-36

**Published:** 2012-04-26

**Authors:** Adrian D Slee

**Affiliations:** 1 School of Life Sciences, Brayford Pool Campus, University of Lincoln, Lincoln, UK

**Keywords:** Chronic kidney disease, Cachexia, Cardiovascular disease

## Abstract

Impaired kidney function and chronic kidney disease (CKD) leading to kidney failure and end-stage renal disease (ESRD) is a serious medical condition associated with increased morbidity, mortality, and in particular cardiovascular disease (CVD) risk. CKD is associated with multiple physiological and metabolic disturbances, including hypertension, dyslipidemia and the anorexia-cachexia syndrome which are linked to poor outcomes. Specific hormonal, inflammatory, and nutritional-metabolic factors may play key roles in CKD development and pathogenesis. These include raised proinflammatory cytokines, such as interleukin-1 and −6, tumor necrosis factor, altered hepatic acute phase proteins, including reduced albumin, increased C-reactive protein, and perturbations in normal anabolic hormone responses with reduced growth hormone-insulin-like growth factor-1 axis activity. Others include hyperactivation of the renin-angiotensin aldosterone system (RAAS), with angiotensin II and aldosterone implicated in hypertension and the promotion of insulin resistance, and subsequent pharmacological blockade shown to improve blood pressure, metabolic control and offer reno-protective effects. Abnormal adipocytokine levels including leptin and adiponectin may further promote the insulin resistant, and proinflammatory state in CKD. Ghrelin may be also implicated and controversial studies suggest activities may be reduced in human CKD, and may provide a rationale for administration of acyl-ghrelin. Poor vitamin D status has also been associated with patient outcome and CVD risk and may indicate a role for supplementation. Glucocorticoid activities traditionally known for their involvement in the pathogenesis of a number of disease states are increased and may be implicated in CKD-associated hypertension, insulin resistance, diabetes risk and cachexia, both directly and indirectly through effects on other systems including activation of the mineralcorticoid receptor. Insight into the multiple factors altered in CKD may provide useful information on disease pathogenesis, clinical assessment and treatment rationale such as potential pharmacological, nutritional and exercise therapies.

## Introduction

Chronic kidney disease (CKD) leading to kidney failure and end-stage renal disease (ESRD) is becoming a global public health problem affecting the United States (US), United Kingdom (UK) and Europe, and other regions in the world; and is linked to poor health outcomes, higher risk of cardiovascular disease (CVD) and mortality [1-8].

CKD is associated with a range of complex deleterious alterations in physiological and metabolic function, such as; worsening and eventual failure of kidney function, accumulation of uremic toxins, termed ‘uremia’, metabolic acidosis, abnormalities in lipid, amino acid, mineral, bone and homocysteine metabolism; malnutrition, insulin resistance, inflammatory and oxidative stress, anemia, vitamin D deficiency, skeletal muscle dysfunction with a reduction in exercise tolerance, and lean body mass (LBM) wasting and ‘*cachexia’*[9-16]. Further, many factors such as dyslipidemia, diabetes and hypertension (traditional CVD risk factors) which coexist within CKD are associated with worsening outcome, an increased risk of CVD and mortality [9]; and potentially other factors, such as inflammatory and oxidative stress, erythropoiten (EPO) resistance and anemia [17], vitamin D deficiency [18] and vascular calcification [19]. Research has also demonstrated the presence of the ‘cardio-renal syndrome’, whereby dysfunction of the kidney and cardiac systems may amplify progressive failure of both systems; i.e. CKD may promote or worsen heart failure and vice versa [17].

It has been generally understood that there is a progressive loss of LBM, muscle wasting and risk of malnutrition as CKD worsens (towards kidney failure) which is thought to be particularly linked to uremia, acidosis, inhibition of normal insulin-IGF-1 anabolic signalling pathways within skeletal muscle and activation of proteolytic systems [12,20]. The loss of LBM and skeletal muscle is a strong predictor of morbidity and mortality and is found to be present within other chronic illnesses, such as; chronic obstructive pulmonary disorder (COPD) and chronic heart failure (CHF). This generalised ‘*cachectic’* response is believed to be associated with chronic activation of the catabolic, ‘proinflammatory acute phase stress response’ and principally related to actions of the proinflammatory cytokines, such as tumor necrosis factor α (TNFα), interleukin-1β (IL-1β), interleukin-6 (IL-6) on central and peripheral tissues [21-25]. Recently, the adipocytokines such as leptin, adiponectin and gut hormones such as ghrelin have also been potentially implicated in the pathogenesis of anorexia-cachexia and metabolic dysfunction (e.g. dyslipidemia) in ESRD [26], as have reported disturbances of normal growth hormone (GH) - insulin-like growth factor-1 (IGF-1) axis activities [27].

The precise relationship between these many factors in kidney disease is being rapidly investigated as the burden of poor health in this patient group is high. Understanding the causal mechanisms involved in the promotion of metabolic dysfunction and risk of CVD and mortality in CKD is critical. Clinicians are recognising the importance of understanding this complex disease, its nutritional implications, assessment and treatment modalities; and the potential to modify health and outcome by pharmacological and non-pharmacological means, e.g. by nutritional therapies [28,29].

## CKD clinical diagnosis, progression and pathogenesis

CKD is defined as either kidney damage or decreased kidney function (*measured by decreased glomerular filtration rate, GFR*) for 3 or more months [1] (see Table 1 for stages of CKD). Kidney damage is defined as pathologic abnormalities or by markers of damage, which is in particular, through the presence of proteinuria/albuminuria. Screening is recommended using ‘untimed urine checks’ for the presence of proteinuria, measuring the total protein-creatinine (>200 mg/g) and/or albumin-creatinine ratio (>30 mg/g as designated cutoff point). Kidney function is measured either using directly measured GFR (mGFR) measuring the clearance of specific exogenous compounds such as inulin or most frequently through the estimation of GFR (eGFR) via serum creatinine levels [30,31]. There are a number of well known eGFR equations which include other factors such as age, sex, body size and race; including the Cockroft-Gault and ‘modification of diet in renal disease’ (MDRD) study equations [30,31]. The MDRD equation is the most well known and used in practice, however its accuracy has been recently debated and a potentially more precise formula has been presented as the CKD-EPI study equation [32,33]. It has also been recognised that methods based solely upon serum creatinine are problematic and may be inaccurate in cases such as in the elderly and in muscle wasting for example, as ageing is associated with a gradual reduction in kidney function and muscle wasting with reduced creatinine synthesis. Therefore, an equation based upon using serum cystatin C has been evaluated [30,31,33]. As in Table 1 CKD progresses through stages 1–5, whereby stage 5 kidney failure is defined as either GFR < 15 ml/min per 1.73 m^2^, which is in most cases accompanied by uremia, or 2) need to commence kidney replacement therapy (dialysis or transplantation), i.e. ESRD. eGFR and albuminuria may act as markers of CVD risk and this has been discussed as to whether they should be incorporated within the traditional Framingham risk factors for CVD to improve risk stratification [34]; although research has found that patients at high risk of CVD, eGFR and albuminuria markers may not aid in risk stratification for CVD but does for renal outcomes [35]. Further, another issue exists whereby CKD is most common in the aged population, but reduced kidney function maybe a normal part of ageing and also older people have a higher risk of CVD despite classification of kidney damage/reduced function [36,37].

**Table 1:** Classification of CKD stages 1–5

Stage	Description	GFR, ml/min/1.73 m^2^
-	At increased risk	≥ 60
1	Kidney damage with normal or increased GFR	≥ 90
2	Kidney damage with mild decreased GFR	60-89
3	Moderately decreased GFR	30-59
4	Severely decreased GFR	15-29
5	Kidney failure	< 15 (or dialysis)

Adapted from [1].

**Figure 1:**
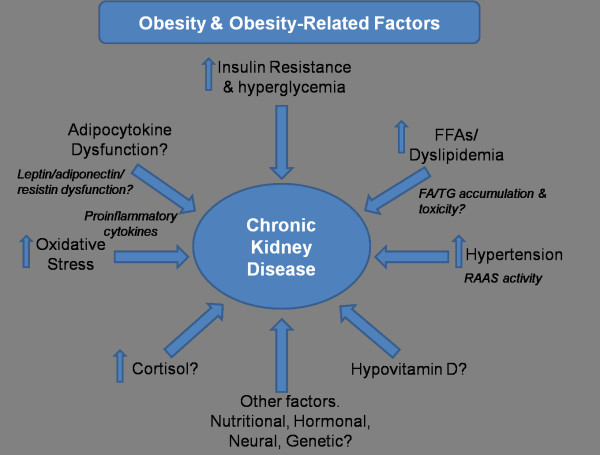
**A range of ‘obesity and obesity-related’ factors have been suggested to be implicated in the progression of chronic kidney disease, CKD.** These include; general insulin resistance and progressive hyperglycemia. Heightened free fatty acids, FFAs common in obesity may lead to further insulin resistance and FA/TG fatty acid/triglyceride accumulation locally within tissues –which may be implicated in cellular dysfunction (e.g. *the promotion of cell death pathways*). Hypertension is common in obesity and metabolic syndrome and may be due to a range of factors including heightened sympathetic nervous system activation and angiotensinogen release from adipose tissue. The hypertension may have a direct damaging effect on microvasculature within renal tissues and via the actions of heightened renin angiotensin aldosterone system, RAAS activity. The dysregulation of adipose tissue-derived adipocytokines such as leptin and adiponectin and proinflammatory cytokine release may strengthen obesity-related factors including insulin resistance, dyslipidemia and oxidative stress. Other factors potentially implicated may include resistin, corticosteroids (*potentially* via *increased adipose tissue production and hypothalamic pituitary stimulation*), hypovitamin D status and other nutritional and genetic factors (e.g. gene polymorphisms). *Note that the precise mechanisms in human in vivo CKD are yet to be fully elucidated, and is speculation at present, based upon association studies, cell and animal studies and pharmacological manipulation.*

The initial development of abnormalities in kidney function has been linked to chronic obesity and insulin resistant conditions, such as the metabolic syndrome (MetS), potentially through a host of ‘obesity-related’ factors (see Figure 1); and these same factors may be therapeutically targeted for reducing/slowing progressive kidney dysfunction [38,39]. Sympathetic hyperactivity, activation of the renin-angiotensin-aldosterone system (RAAS), and the related development of hypertension are implicated; and this may be further aggravated by excess dietary salt intake and effects of insulin resistance and hyperinsulinemia on sodium retention. [2,40,41]. The abnormal production of ‘adipocytokines’ (adipose tissue-derived cytokines), have been potentially implicated as playing a role [42] and dysregulation of glucocorticoid activities may be implicated in metabolic dysfunction [43]. The progression of metabolic and cellular dysfunction both systemically and locally within kidney tissue is linked to many diverse and complex pathways currently being elucidated; these include factors mentioned, and in particular the heightened production of proinflammatory cytokines, IL-1β, -6, -8, TNFα and interferon gamma (IFNγ), oxidative stress and abnormalities in glucose and lipid metabolism (e.g. impaired glucose tolerance, glycemia and dyslipidemia) (see Figure 1). The dyslipidemia associated with worsening CKD and in ESRD is also a contributing factor in metabolic dysfunction, CVD risk and glomerulosclerosis [14,16]. Specifically, reduced clearance of triglyceride-rich lipoproteins and reduced high-density lipoprotein production and levels are common in ESRD [14,16].

Further, endothelial dysfunction which is common in CKD and promotes or accelerates atherosclerosis is currently hypothesised to be due to a combination of the factors described above including; hypertension but also in particular, oxidative stress, insulin resistance, depressed nitric oxide synthesis, adipocytokine dysfunction and hemodialysis-induced factors (in ESRD) [44].

The effects of CKD on protein metabolism and dietary treatment is complicated and beyond the scope of this review; however as CKD develops and kidney function progressively worsens towards kidney failure the ability to process and efficiently clear waste products of protein metabolism decreases. As GFR decreases dietary protein nitrogen intake must be reduced. Although debated, a very low protein diet is a standard dietary therapy for patients to delay kidney pathogenesis [12,28,29]. This dietary requirement may have beneficial effects through reducing uremic toxins, but also detrimental effects through lowering plasma amino acid levels and negatively impact upon protein turnover, i.e. by stimulating a net loss of protein in LBM and skeletal muscle tissue. The process of dialysis itself may also interfere with protein metabolism and increase protein catabolism [45]. Bammens *et al.*, 2004 found that small intestinal protein assimilation (digestion and absorption) was significantly reduced in ESRD patients on dialysis [45]. However, haemodialysis has also been found to improve insulin sensitivity [46].

## The acute phase response, proinflammatory cytokines and metabolism

During the development and pathogenesis of CKD as with many other chronic disease states a typical systemic and local physiological immunologic stress response and activation of an *acute phase response* takes place [21,47,48]. There is characteristic activation of a proinflammatory stress reaction with a reprioritisation of metabolic and immune activities, principally directed and orchestrated by the cytokine peptide mediators. Protein synthetic priorities tend to be heightened in hepatic and visceral tissues with a simultaneous depression of anabolic activities within skeletal muscle. Protein catabolism is increased peripherally supplying a raised demand for amino acids in the liver for the production of acute phase proteins and reactants (APRs), glutathione, and glucose (*gluconeogenesis*). In addition, an insulin resistant state develops and carbohydrate and lipid metabolism is altered favouring mobilisation of stores (*glycogenolysis* and *lipolysis*). Specific alterations to endocrine function are noted which aids in inducing the insulin resistant ‘mobilisation state’, including an increase in glucagon, glucocorticoids, catecholamines and GH release (although simultaneous depression of anabolic effects of the GH- IGF pathway, especially within skeletal muscle) [47,48]. In CKD and particularly in kidney failure other endocrine abnormalities are notable including thyroid dysfunction, hypogonadism [49] and GH-IGF resistance [27] which promotes the catabolic state (*and the depression of anabolism*).

Specific cytokines have been identified with proinflammatory actions, including IL-1β, IL-6, TNFα and IFNγ which increase during inflammatory stress and have modulating effects on appetite (induce anorexia), carbohydrate, lipid and protein metabolism (e.g. increasing insulin resistance, lipolysis and proteolysis) [47,48]. A key feature in chronic disease states such as CKD includes cachexia and lipid abnormalities; and dyslipidemia is thought to be due to the effects of chronic activation of hormone-sensitive lipolysis and a reduction in triglyceride catabolism by cytokines [21].

Further, proinflammatory cytokines increase the production of reactive oxygen species (ROS) and are themselves regulated in a positive feedback loop via the nuclear factor kappa beta (NFkB) pathway [50]. Therefore the cellular damage caused by the pro-inflammatory response stimulates the ROS production cycle and in turn activating further cytokine production; hence chronic activation as observed in chronic illness such as advanced CKD may become damaging, potentially having deleterious effects on health.

## Inflammatory markers in CKD and clinical outcome

Studies have suggested that the levels of cytokines, IL-1β, IL-6 and TNFα and hepatic acute phase proteins such as serum albumin (S-Alb) C-reactive protein (CRP) and fibrinogen may be helpful in the clinical assessment of patients. For example, CRP which is a strong indicator of systemic inflammatory status, when measured by a high sensitivity assay (hsCRP), may also act as an indicator of CVD risk and cardiovascular events such as myocardial infarction and stroke [51-53].

A relationship exists between malnutrition, inflammatory markers with CVD risk, clinical outcome and mortality rates- with particular relevance in stage 4/5 and kidney failure. Long term mortality prediction was recently evaluated in a 5-year prospective study in 42 ESRD patients [54], and the most robust markers found were CRP and S-Alb. Stenvinkel and Lindholm, 2005 discuss the relevance of CRP and IL-6 in predicting CVD and mortality and the potential of IL-6 as acting as a good predictor [55]. Honda et al., 2006 found IL-6 best predicted CVD in ESRD patients (*n* = 176), and that S-Alb, IL-6 and fetuin A (a circulating calcium-regulatory glycoprotein that inhibits vascular calcification) best predicted mortality, but interestingly not hsCRP [56]. It should also be noted that new research has identified that IL-6 has mixed pro/anti-inflammatory activities and that although IL-6 levels correlate a worse outcome and mortality in human studies; it is also produced locally within skeletal muscle (as a ‘myokine’) during exercise and may exhibit beneficial metabolic effects [57]. Further studies will be required to evaluate whether hsCRP and IL-6 in this patient population group are robust markers of inflammation and disease severity and whether they give an *indication* or predict clinical risk or outcome.

Studies have also determined associations between higher levels of inflammatory markers such as CRP and measures of cardiac autonomic nervous system function, including lower heart rate variability (HRV). Lower HRV measures tends to indicate heightened sympathetic activities and reduced parasympathetic vagal activities, is associated with worse patient outcome and CVD risk in a number of human disease states [58]. For example, strong associations exist between hypertension, diabetes and decreased HRV. Research is necessary to evaluate this relationship within CKD and inflammation, its value, and validity as a prognostic marker.

The proinflammatory cytokines also have a negative impact on other important factors in CKD such as EPO sensitivity and hence may act as a secondary factor (other than reduced kidney EPO production in progressive CKD) promoting risk of anemia in CKD patients [17].

## Anorexia-cachexia in CKD

In many chronic disease states and typically in CKD there is a characteristic reduction in food intake (anorexia) and increase in resting energy expenditure (REE), coupled with increased net protein breakdown and a progressive loss of LBM and skeletal muscle [59,60]. Termed the ‘anorexia-cachexia’ syndrome (ACS), this is thought to be principally driven by proinflammatory cytokines and their effects peripherally on skeletal muscle pathways regulating protein turnover and centrally within hypothalamic neurons regulating appetite [59,60]. With respects to the specific loss of muscle in CKD, this is thought to be driven by many factors such as the cytokines, with concomitant upregulation of the NFkB pathway, and genes involved in the ubiquitin proteasome protein degradation system. Further, insulin/IGF-1 resistance and up-regulation of glucocorticoids (discussed in later sections) may further support an increase in protein degradation and decrease in protein synthesis. Myostatin may be upregulated leading to reductions in Akt phosphorylation, downstream reductions in protein synthetic (mTOR), myogenic (pax3, MyoD) pathways and upregulation in caspase-3 and components of the ubiquitin proteasome system (12, 20, 21, 60). A range of other factors implicated include acidosis, uremia, dialysis treatment and other mediators mentioned later in this review.

It is thought that there may also be a perturbation in the normal regulation of appetite, energy intake and energy expenditure (hence leading to negative energy balance) in CKD. Although the mechanisms are not completely understood it is thought that cytokines (e.g. IL-1β, IL-6 and TNF α) participate in developing this state; and that levels/activities of leptin (an *anorexigenic* peptide reducing food intake) and ghrelin (*orexigenic* peptide increasing food intake) may be disturbed. Normally, central hypothalamic neurons producing melanocortins (proopiomelanocortin (POMC)/cocaine- and amphetamine-regulated transcript (CART) neurons) activated by leptin have an anorectic and catabolic biological effect whereas the neuropeptide Y (NPY) and agouti-related protein (AgRP) expressing neurons activated by ghrelin have a downstream orexigenic effect. These neuronal appetite pathways may also cross-regulate one another. In ACS, the hypothalamic response to peripheral signals such as circulating leptin and ghrelin may be dysfunctional with persistent activation of the anorexigenic POMC/CART neuronal systems, and reduced activation of NPY/AgRP with subsequent effects of increasing REE and reducing appetite. However, in CKD as will be described in the following sections, the levels of leptin and ghrelin are generally *increased* which is unique to this disease state [21,60,61].

Heightened inflammation and presence of ACS tends to be common characteristics of the CKD patient population and in particular in stage 5 kidney disease. For example, in ESRD dialysis patients, poor nutritional status and appetite has been associated with a heightened inflammatory state [62].

The effects of CKD on measured REE are somewhat complex as instinctively it may be expected that REE would increase with the severity of kidney dysfunction and inflammatory stress. This may not always be the case in all patient observations and is difficult to accurately assess. Avesani et al., 2004 demonstrated that REE is not influenced significantly by kidney function, but possibly by inflammatory status, e.g. by measuring serum levels of APRs and pro-inflammatory cytokines [63]. Further, energy expenditure may appear within normal range or decrease in some patients due to the simultaneous reduction in LBM and habitual physical activity which is both common and a characteristic of the condition. Ultimately, this ACS state affects clinical outcome as outlined in a recent study by Yen et al., 2010, in which 959 taiwanese ESRD hemodialysis patients were followed over a three year period [64]. They found after analysis that those patients with lower body mass index, BMI levels (<18.5 kg/m^2^) suffered a significantly higher mortality rate to other patients with normal, overweight and obese classifications; and hence may be considered as a ‘*risk factor paradox’* when comparing against obese patients.

## Other potential mediators of metabolic dysfunction in kidney disease

A number of other mediators and hormonal systems may become perturbed in CKD and promote metabolic dysfunction, ACS and affect health. There are numerous nutritional, metabolic and hormonal factors however focus will be directed in this review on the following; the GH-IGF-1 axis, ANG II and aldosterone, adipocytokines; leptin, adiponectin; ghrelin, vitamin D and glucocorticoids.

## The GH-IGF-1 axis

The Growth Hormone (GH) - Insulin-like Growth Factor-1 (IGF-1) axis is a major controller of cell and tissue growth and development in human, stimulating protein synthesis in a range of tissues. Stimulation of GH production from the pituitary gland by stimuli such as exercise, stress and sleep leads to increases in pulsatile output from the pituitary, leading to increased activation of hepatic GH receptors and IGF-1 production. IGF-1 is a key peptide involved in cell growth and protein turnover acting as the primary mediator of many of the responses regulated by GH in tissues [65,66]. IGF-1 is secreted systemically (hepatic), locally and recently a muscle specific IGF isoform called ‘*mechano growth factor’* (MGF) has been identified. IGF-1 possesses glucose-disposal, anti-apoptotic and anti-proteolytic activities in muscle, and shares some cell signalling pathways with insulin. Plasma IGF-1 levels have been used as a potential measure of nutritional status and ‘anabolic responsiveness’ and the association exists such that during malnutrition IGF-1 reduces and during nutritional excess increases in proportionality [67,68].

GH and IGF-1 also have major effects on kidney growth, structure and function and their overall activities are reduced in patients with kidney disease which may have a negative impact on kidney function and affect pathogenesis [69]. In bone and muscle tissue as the GH-IGF axis controls and regulates growth and development, reductions in activities may potentially accelerate cachexia-type phenomena in CKD. Evidence in children with CKD is startling in that pronounced alterations in the GH-IGF axis occur with a characteristic induction of GH resistance and stunting/retardation of normal growth; and a high potential for poor patient outcome [70].

The mechanism of this reduction and inhibition of GH-IGF activity is likely through CKD-disease specific and non-specific factors. The effects of chronic neuro-endocrine stress has a significant effect on down-regulating anabolic hormone systems such as the GH-IGF-1 axis in humans [71]. This is characterised by the effects of heightened activities of hypothalamic corticotrophin-releasing hormone (CRH) and pituitary adrenocorticotropin hormone (ACTH) increasing adrenal cortisol production. In addition, a reduction in food intake and nutritional status can reduce IGF-1 activity [67,68], which is common in CKD patients with malnutrition/ACS. However, further mechanisms of inhibition of the GH-IGF-1 system have been proposed including the effects of chronic uremia attenuating GH receptor-JAK2-STAT signal transduction pathways [27,72]. Uremia itself and acidosis is understood to increase cortisol levels in kidney dysfunction. Other plausible negative factors which may antagonise or inhibit activity of the GH-IGF-1 system in CKD include the pro-inflammatory cytokines at a peripheral cellular level (e.g. skeletal muscle) and through reinforcement of hypothalamic neuro-endocrine stress [27,73].

This may mean that uremic malnourished patients are likely to have long-term reductions in GH-IGF activities systemically affecting tissues such as skeletal muscle. Another known factor includes induction of insulin resistance which is typically increased during disease states characterised by chronic inflammatory stress [47]. Insulin resistance has been found to be higher in critically ill patients with acute renal failure (ARF) [74]. This is expected, however, patients with higher insulin resistance had significant alterations in IGF-1 pathway activity (IGFBP-3 was significantly lower and IGFBP-1 higher) and mortality. Interestingly, Lee et al., 2007 found that in non-diabetic ESRD patient’s (*n* = 21) insulin resistance (measured by HOMA-IR) correlated with muscle wasting (LBM measured by DEXA), and a marker of muscle protein degradation (14 kDa actin sampled from rectus abdominis tissue) potentially indicating the importance of insulin-sensitive anabolic/anti-catabolic pathways in the regulation of protein turnover in skeletal muscle in ESRD [75].

Further, Abdulle et al., 2007 found that low circulating IGF-1 levels strongly correlated with higher cardiovascular risk (using measurements of systolic and diastolic blood pressure) in adult CKD patients [76]. This may indicate that IGF-1 may also be a novel indicator of CVD risk in this patient group; or it may be that the low IGF-1 generally relates to poor nutritional status; and hence worse clinical outcome, further evidence will be required.

In summation, the GH-IGF axis may be an important pathway for further investigation in human CKD and in particular stage 4/5 patients, experiencing uremia, inflammation and malnutrition-both as a potential means of assessment of nutritional status/anabolic activity and of treatment. The use of recombinant human GH (rhGH) and rhIGF-1 in CKD patients is an obvious choice with some potential to improve muscle protein turnover and nitrogen retention. In children with CKD and growth retardation use of GH and IGF-1 has demonstrated to have positive effects on growth and outcome [70] and recently a newer GH releasing hormone ‘super-agonist’ has been utilised in stage 4/5 CKD patients showing significant positive effects on anabolism and LBM [77].

## The renin-angiotensin aldosterone system and angiotensin II

The renin-angiotensin aldosterone system (RAAS) plays an important role in the regulation of blood pressure control and body fluid homeostasis [41,78-82]. Angiotensin II (ANG II) is the main effector peptide of the RAAS, with potent hypertensive actions and has been implicated in vascular remodelling and pathogenesis in cardiac and renal tissues. Its actions are mediated through the ANG II receptors type 1 and 2 (ATII-1 and −2 R). ATII-1R has been associated with vasoconstriction, sodium re-absorption and growth promotion, where as the ATII-2R with opposing actions. ANG II contributes to atherogenesis through increasing oxidative stress (reactive oxygen species, ROS) and the expression of pro-inflammatory genes via the NF-kB pathway [83,84]. Aldosterone is the principal mineralcorticoid secreted from the adrenal glands in response to circulating ANG II, adrenocorticotropin hormone (ACTH) from the pituitary gland and extracellular potassium ions [41,81,82]. Aldosterone has ‘genomic’ and ‘non-genomic’ actions through the cytosolic mineralcorticoid receptor (MR) and functions both independently and in concert with ANG II cell signalling pathways, promoting ROS production and tissue fibrosis (partly via the epidermal growth factor (EGF) receptor) [41,80,81].

CKD is typically associated with the presence of hypertension which increases the risk of cardiovascular events and kidney damage. Inhibition of ANG II through the use of pharmacological ATII-1R blockers (ARBs) or angiotensin-converting enzyme inhibitors (ACEIs) is used as common therapy for hypertension, including within CKD patients [85]. Use of these pharmacological agents has been shown to have positive effects on patient health and outcome, such as the delaying of progression to stage 5 kidney disease, having a ‘reno-protective’ type action, which was suggested to be most likely through a consistent reduction in blood pressure [85], although there may be other protective metabolic actions of ANG II blockade [86]. MR receptor blockers/aldosterone antagonists have been utilised as an additional therapy to ARBs/ACEI in cardiac disease and recently in CKD due to potential effects on reducing proteinuria and kidney damage; and to a lesser extent on blood pressure. [82,87].

Recent evidence suggests that ANG II and aldosterone may have significant metabolic effects and may contribute to the development and progression of insulin resistant conditions such as obesity, metabolic syndrome and cachexia [41,81,87-90]. ANG II has been found in studies to induce insulin resistance via activation of ANG II-induced protein tyrosine phosphatase activation leading to dephosphorylation of the insulin receptor [89-91]. Aldosterone acting via the MR is thought to interact with ANG II and ROS signalling leading to activation of redox-sensitive kinases known to phosphorylate and inactivate insulin receptor signalling molecules, such as IRS-1 [41,81].

ANG II has also been linked to promoting dyslipidemia in studies using chronic infusion of ANG II in rat models [92]. Kouyama *et al.*, 2005, demonstrated that mice lacking the ATII-1aR exhibit an attenuation of diet-induced weight gain and adiposity through increased energy expenditure [93]. In addition, ANG II modulated adipocytokine production via the ATII-1R. The relationship between the RAAS system and insulin signalling pathways has led to the hypothesis that ANG II blockade (through use of ACEI and ARBs) in insulin resistant conditions such as the metabolic syndrome would be of benefit to metabolic and cardiovascular health [94]. Interestingly, Zandbergen *et al.*, 2006 observed improved insulin sensitivity and raised IGF-1 levels when administering losartan (an ARB) to patients with impaired fasting glucose [95]. Studies in patients with CKD are promising and demonstrate the beneficial effects of reducing ANG II activity on metabolic and inflammatory function. For example, de Vinuesa et al., 2006 demonstrated the use of the ARB olmesartan (40 mg daily for 16 weeks duration) in 52 adult patients showing typical reductions in systolic and diastolic blood pressure, but also significant reductions in proteinuria, glucose, insulin, insulin resistance (HOMA-IR index), glycated haemoglobin, hsCRP and fibrinogen [96].

Additionally, ANG II has been observed to induce skeletal muscle wasting, down-regulate systemic and autocrine IGF-1 pathways and increase muscle protein degradation in rats [97]. Song *et al.*, 2005, demonstrated that ANG II can down-regulate muscle-specific IGF-1 pathways and activate caspase-3, the pro-apoptotic intracellular protease [98]. Further to this, Burniston *et al.*, 2005 reported that ANG II induces skeletal and cardiac muscle cell apoptosis in rats [99]. Recently, aldosterone has also been thought to play a role in regulating muscle function (in particular cardiac) and MR antagonism via drugs such as spironolactone has been suggested as a means of reducing the decline in skeletal muscle function and apoptosis in age-related sarcopenia [88]. If proven efficacious, there may be potential to reduce muscle wasting in human CKD; with particular interest in down-regulating the effects of chronic RAAS activation on insulin/IGF pathway inhibition and ROS generation.

## Adipocytokines

Adipose tissue is a highly active endocrine organ playing an important role in the clearance and storage of fatty acids, the regulation of energy homeostasis/metabolism, appetite control, insulin function and inflammatory activities; and has been suggested to be implicated in disease progression and metabolic dysfunction [100-105]. Studies demonstrate that in models of absent, reduced or dysfunctional adipose tissue, e.g. *lipodystrophy,* patterns of ectopic lipid accumulation in hepatic and skeletal muscle tissues, lipotoxicity and insulin resistance develop. There is evidence that in these extreme forms of insulin resistance, such as in lipodystrophies and in the MetS there are clinical observations of kidney disease [39,105].

Adipose tissue secretes a large number of peptides (*adipocytokines*) with autocrine, paracrine and endocrine activities. Peptides including leptin, adiponectin, resistin, TNF-α, IL-6 and components of the RAAS such as angiotensinogen, ACE and ATII-1R (others include VEGF, MCP-1, RBP-4 and TIMP-1 and the MR). Studies suggest that a positive correlation exists between increased adipose tissue/fat mass and systemic inflammatory markers [106]. It should be noted that adipose tissue also contains active macrophages and immune cells that secrete pro-inflammatory cytokines. Hence, there is a continuing debate in the literature as to the link between dysfunctional adipose tissue (e.g. as in obesity) and/or raised proinflammatory adipocytokine patterns and systemic inflammation, insulin resistance and cellular dysfunction [100,107,108]. The role played within CKD pathogenesis has also yet to be identified. Axelsson *et al.*, 2004 observed a positive relationship between truncal fat mass and inflammation in ESRD patients, and a strong correlation between markers of inflammation and an atherogenic lipoprotein profile [109].

Key adipocytokines currently being intensely investigated include leptin and adiponectin. Leptin has been extensively studied in animal models and humans. Resistin, is lesser well known and is a 108 amino acid peptide shown to have some effects on inflammatory activities and insulin resistance in animal models; however in humans its function remains controversial.

## Leptin

Leptin as previously discussed is an adipocytokine with well studied characteristics in humans. Leptin levels rise in response to increased energy deposition as fat mass and in obesity. This characteristic signal acts centrally in hypothalamic neurons to decrease energy intake and increase energy expenditure, which also involves raising sympathetic activity and peripheral actions of leptin on skeletal muscle activating AMP-activated protein kinase, (AMPK). During obesity it is thought that central hypothalamic resistance to leptin, similar to insulin resistance may take place. Further, leptin may have immuno-regulatory and proinflammatory actions as recognised in obesity and disease states [110]. Maachi et al., 2004 demonstrated in obese nondiabetic women that leptin levels are associated with fat mass and local and systemic markers of inflammation such as CRP [106].

In CKD the picture is more complicated as the initial development of CKD may be with a background of obesity; and hence raised leptin levels (*and leptin resistance*). During CKD disease progression (*and potentially ACS*), research shows that hyperleptinemia is observed in humans, due to reduced renal clearance of leptin and that this is associated with concomitant inflammation and loss of LBM [26]. This abnormal response may play a role in the development of the ACS, in particular anorexia, overall metabolic dysfunction and may affect patient outcome. In this scenario research into the use of treatment to reduce leptin activities is warranted and ongoing.

## Adiponectin

Adiponectin is reported to modulate lipid and glucose metabolism and insulin sensitivity, partially through activation of AMPK [111]. Adiponectin exhibits anti-diabetic, anti-inflammatory and anti-atherogenic effects and hypoadiponectinemia is associated with conditions such as insulin resistance, obesity, type II diabetes (early stages) and dyslipidemia. Interestingly, mixed reports during different stages of CKD and kidney failure have appeared, demonstrating paradoxically, an up-regulation in adiponectin in kidney failure [111-116]. Further, Lin, Hu and Curhan, 2007, found in 733 men with type II diabetes that higher serum adiponectin concentrations were associated with a reduced odds of moderate kidney dysfunction [117].

Thiazolidinediones (TZDs) have been shown to act as insulin sensitizers and used as therapeutic agents for the treatment of dyslipidemia and type II diabetes [118], both common occurrences in CKD. Their proposed modes of action are through the activation of the peroxisome proliferator-activated receptor γ (PPARγ) and through the up-regulation of mediators such as adiponectin [111,119]. Despite recent recommendations for TZD treatment being re-examined in the light of potential increases in adverse cardiovascular events, previous trials have shown beneficial effects of TZD treatment in CKD patients [120]. Schneider et al., 2008, describes how pioglitazone treatment in CKD-defined patients was shown to improve overall CKD patient outcome reducing cardiovascular events and death in the ‘PROspective pioglitAzone Clinical Trial In macro Vascular Events’ (PROactive) trial [120].

The relationship between adiponectin, ANG II blockade, TZD treatment and its physiological effects in human CKD is intriguing and requires an indepth examination of wider literature. Studies by Clasen *et al.*, 2005 demonstrated in cell and rat models that PPARγ-activating ATII-1R blockers induce adiponectin production [121]. In addition, Diep *et al.*, 2002, demonstrated in ANG II infused rats that TZDs attenuate hypertension, normalize cell growth, improve endothelial function and prevent the up-regulation of ATII-1R, cell cycle proteins and proinflammatory mediators [122]. In humans, Furuhashi *et al.*, 2003, observed that blockade of ANG II activities using either an ARB or ACEI increased adiponectin in essential hypertensive patients [123]. However, in patients with CKD the picture is somewhat different and baseline levels of adiponectin tend to be higher and the effects of ARB medication different. De Vinuesa, 2006 found no significant effect of ANG II blockade with olmesartan on either leptin or adiponectin despite increases in insulin sensitivity [96]. A recent study by Guo et al., 2009 suggested again a paradoxical response in type 2 diabetic nephropathy CKD patients (CKD stages 1–4) [124]. At baseline all CKD patients exhibited high adiponectin values positively associated with insulin and insulin resistance. Subsequent administration of the ARB losartan (100 mg daily for 6 months) compared to a control CKD group on amlodipine found significant reductions in adiponectin (*P* < 0.01) accompanied by reductions in HbA1c (*P* < 0.01), fasting insulin (*P* < 0.01) and HOMA-IR, measure of insulin resistance (*P* < 0.01). The relative increase in adiponectin observed in CKD patients and its apparent reduction following ARB administration at present has an unknown effect through an unknown mechanism. This effect may or may not be beneficial as described by Guo et al., 2009, and may be due to alterations in kidney function (e.g. clearance of adiponection) and/or the development of adiponectin resistance in CKD (and subsequent reversal following ARB administration) [124].

## Ghrelin

Ghrelin is a 28-amino acid peptide synthesised and secreted principally in the stomach and is an endogenous ligand for the growth hormone secretagogue receptor (GHSR), stimulating GH release. A number of reviews have highlighted its effect on appetite, energy expenditure and body weight regulation [102,125,126]. Ghrelin stimulated appetite through activation of NPY neurons in the hypothalamus and levels tend to be raised in between and prior to meals.

Ghrelin levels have been found to be raised in catabolic and wasting disorders. Ayala *et al.*, 2004 found markedly elevated ghrelin levels in advanced kidney failure which also correlated well with levels of fat mass, plasma insulin and serum leptin levels [127]. Pérez-Fontán et al., 2004 found that plasma ghrelin levels were increased in haemodialysis and peritoneal dialysis patients [128]. In addition, there was a correlation found between low dietary intake and plasma ghrelin. Wynne et al., 2005, demonstrated that subcutaneous ghrelin administration enhanced food intake in peritoneal dialysis patients with mild and moderate malnutrition which further stimulated interest in this peptide [129].

The relevance of these observations requires examination as CKD is a catabolic disease with cachexia and anorexia as key features. Increases in ghrelin may act in a counter-measure fashion in an attempt to increase appetite and GH-IGF anabolic activity which are both down-regulated in this disease in human. Another hypothesis similar to the effects of CKD on other hormonal axis may be that down-regulated ghrelin activity or relative ghrelin resistance, potentially due to inflammatory stress and uremia takes place. Further, reduced degradation and clearance of ghrelin by the kidneys is another plausible factor. A review article by Cheung and Mak, 2010 highlights the relevance and importance of ghrelin in CKD and its potential benefits as a pharmacological treatment in improving appetite, body composition (anti-cachectic therapy) and potentially decreasing inflammation (reduction in circulating cytokines) [61]. They argue that increased ghrelin levels in dialysis patients have not been fully evaluated and observations are likely due to the inappropriate use of detection techniques not sensitive to the different forms of ghrelin, i.e. the two major forms of ghrelin; acyl-ghrelin and des-acyl ghrelin. Further, that des-acyl ghrelin may be the form which is elevated and may actually have a reverse effect to acyl-ghrelin, promoting anorexia rather than stimulating appetite [61]. Future research requires a rigorous examination of techniques of ghrelin detection in CKD and greater overall systematic data accumulation and analysis in human patients. Regarding the theoretical effects of ghrelin administration in CKD patients, its relevance and potential utilisation is yet to be understood in adult human and childhood CKD. In adult human CKD patients long term studies need to be performed as short-term ghrelin administration has been shown to improve food intake without affecting energy expenditure which is positive. Studies in rat models of uremic CKD such as by DeBoer et al., 2008 show ghrelin administration causing improvements in food intake, LBM, decreases in muscle protein degradation and circulating inflammatory cytokines [130].

## Vitamin D

CKD is associated with a host of disorders of mineral and bone metabolism termed CKD-metabolic bone disease, MBD (10, 11) which is typically characterised by hyper-phosphataemia, hypocalcemia, secondary hyperparathyroidism and decreased synthesis of active vitamin (vit) D (1,25(OH)D/1, 25-vit D). Recently vit D status has been found to be of importance in CKD. Vit D is an essential nutrient found in a range of foods such as dairy products and fish, and dietary supplements as vit D_2_, ‘ergocalciferol’ and vit D_3_ or ‘cholecalciferol’. Vit D_3_ is mainly generated by ultraviolet UV-B radiation on skin tissue and acts as a major source of vit D [131,132]. Both vit D_2_ and D_3_ are inactive precursors which are metabolised within the liver via hydroxylation (25-hydroxylase enzyme) to 25-hydroxyvit D, (25(OH)D/25-vit D) [105]. Classically, it has been assumed that 25-vit D is an inactive precursor which becomes activated via a 2^nd^ hydroxylation step within kidney tissue (1α-hydroxylase enzyme) to become 1,25-vit D, the active compound involved in the regulation of calcium, phosphorous and bone metabolism [131]. Research has indicated that vit D has ‘hormone-like’ characteristics regulating a whole range of different organs, tissues and processes including; immune, skeletal muscle and cardiovascular function [131,133]; and that 25-vit D may have functional activities itself, able to activate the vit D receptor (VDR), although with less affinity. Evidence also suggests that extra-renal 1α-hydroxylase enzyme activity is present in a range of tissues including vascular, immune and gastrointestinal cells [133]. Further, that reduced vit D status due to a combination of decreased sunlight exposure and dietary intake is potentially associated with an increase in CVD risk [132].

In CKD, vit D status is usually impaired with levels decreasing in relation to worsening of kidney function. This may be due to a combination of a reduction in production of 1, 25-vit D from kidney tissue, lowered vit D intake during restrictive dietary therapy in CKD and decreased sunlight as the disease state worsens (e.g. reduced physical mobility) and other unknown deleterious factors. Reduced kidney function and active vit D status may in turn leads to disturbances in normal calcium and phosphate metabolism, bone turnover and potentially hyperparathyroidism in CKD-MBD. This may lead to increased fracture risk, especially in those who are older and with worse nutritional status, e.g. those suffering from wasting and forms of CKD-related malnutrition. In respects to CVD risk, outcome and mortality, evidence is emerging that vit D status may be a non-traditional risk factor for CVD and may have been previously under-estimated [18]. Barreto et al., 2009 measured the prevalence of vit D deficiency in 140 CKD patients (stages 1–5) and found that 42% were suffering from deficiency (25-vit D: ≤ 15 ng/ml), 34% from insufficiency (25-vit D: 16–30 ng/ml); and those with ≤ 16.7 ng/ml having a significantly lower survival rate compared to other patients [134]. Garcia-Canton et al., 2010 evaluated the association between vascular calcification and (25-vit D) in 210 CKD patients (stages 4–5) [135]. The study found that only 18.5% of patients had adequate levels of 25-vit D (>30 ng/mL) and that there was an association with reduced vit D status and vascular calcification. In the Barreto et al., 2009 study the relationship between patient survival and vit D was not affected by calcification score; and hence vit D was an independent risk factor [134].

Potential mechanisms of increased CVD risk may include lack of control of calcium and phosphate metabolism and other unknown vit D-dependent mechanisms, including; reduced regulation of anti-atherogenic processes, reduced inhibition of cardiac hypertrophy and regulation of the RAAS. In a review article by Levin and Li, 2005, they describe studies which demonstrate improved outcome in dialysis patients undergoing vit D therapy [18]. In contrast, a review by Mizobuchi et al., 2009 describes conflicting studies where vit D analogues may actually increase vascular calcification in animal and cells models, however, they conclude that direct evidence in human CKD patients is lacking and that low serum 1, 25-vit D levels are associated with vascular calcification within the general population and in CKD; and that vit D analogues improve patient survival [19].

Vit D may play a significant role in a whole range of other functions within the body including, the maintenance of skeletal muscle function, proliferation and differentiation and immune function [131,136,137]. Further, a relationship between vit D and type 2 diabetes and inflammation has been suggested [138]; however, further studies will need to be undertaken and to evaluate in detail the roles and the effect of vit D deficiency, sufficiency and high-dose replacement within the CKD population.

## Glucocorticoids

Glucocorticoids and their role in metabolic dysfunction during clinical disease has a strong research base; and they may play a role in the development of metabolic and kidney dysfunction in CKD. Hypercortisolemia is a characteristic of many inflammatory stress and disease states and is known to increase insulin resistance, protein catabolism, gluconeogenesis and down-regulate anabolic pathways such as GH-IGF-1 activities and androgens; and aid in inducing LBM wasting [43,47].

Glucocorticoids have been implicated in the pathogenesis of the obesity-associated MetS with a role in the development of hypertension, insulin resistance, glucose intolerance and dyslipidemia [43]. Evidence has demonstrated that cortisol levels and glucocorticoid activities are increased during obesity states and maybe due to increased hypothalamic-pituitary-adrenal activity (and sympathoadrenal stress) and adipose tissue-derived cortisol. In addition, cross-talk and receptor activation of the MR and GR by cortisol and aldosterone may also heighten activities [41]. Levels of corticosteroid binding globulin (CBG) may also be modified increasing active cortisol; and enzyme activities of the 11β-hydroxysteroid dehydrogenase isozymes (11β-HSD) 1 and 2 affecting ratios of active cortisol: inactive cortisone. As isoforms exist within tissues such as kidney, adipose and liver affecting activity/inactivity, the relationship between MetS and kidney disease pathogenesis requires investigation.

In respects to hypertension the glucocorticoids may promote hypertension through actions on kidney and endothelial cells and increasing production of angiotensinogen from adipose tissue (and concomitant rise in RAAS activation), and MR activation- although the precise mechanisms are yet to be elucidated [41,43,140]. This may play a role in kidney pathogenesis in chronic hypercortisolemic states. A recent review by Smets et al., 2010 provides convincing evidence for cortisol (or hypercortisolemia) to negatively impact upon kidney function [139]. Further, evidence is also present suggesting that in CKD clearance and excretion of cortisol is impaired (which may be expected), which can increase half-life and serum levels. These activities may be potentiated in CKD by co-activation of the RAAS system and in particular aldosterone having combined negative effects including promotion of cellular insulin resistance and muscle protein loss. The potential dysregulation in cortisol metabolism and effects on metabolic dysfunction, hypertension and pathogenesis in CKD warrants further attention and investigation. Pharmacological modification of these responses in disease also needs to be explored as for example with use of specific 11β-HSD 1 inhibitors to reduce systemic cortisol activities [43] and additional MR-blockade [88].

## Summary

**Figure 2:**
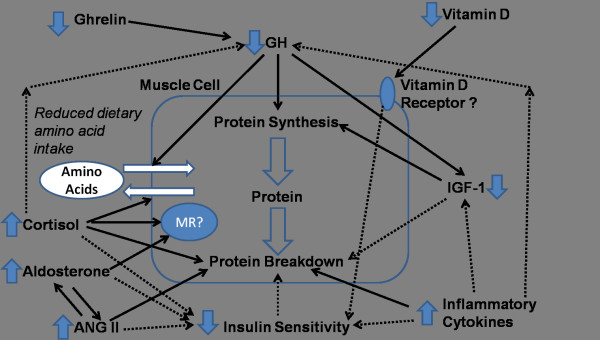
**Depiction of some of the different pathways that may potentially interact in muscle tissue in CKD affecting protein turnover.** Using the example of muscle tissue it can be observed that CKD causes perturbations in a range of factors which are involved in muscle metabolism and protein turnover. Firstly, CKD and uremia causes a decrease in food intake, anorexia by multiple mechanisms including the possible reduction in active ghrelin. Further, therapeutic low protein diets reduce dietary protein intake further and potentially the amino acid pool within muscle. The reduction in dietary intake and amino acids is likely to have a detrimental effect on both direct protein synthetic pathways within muscle (and suppression of protein breakdown, e.g. branched-chain amino acids, BCAAs) but indirectly through insulin and the GH-IGF-1 axis. The GH-IGF-1 axis is further down-regulated in CKD possibly through direct feedback control at the hypothalamus-pituitary level (i.e. via increased corticosteroids and cytokines, and reduced ghrelin) and at a cellular level of GH resistance (potentially via effects of cytokines). GH-IGF-1 has potent effects on amino acid transport, protein synthesis and suppression of protein breakdown (via IGF-1). Insulin resistance is common in CKD and as with other chronic diseases which are characterised by a proinflammatory response. This usually has a general metabolic effect and a local effect in muscle with a reduction in nutrient transport (glucose and amino acids)/responsiveness of the cell and reduction in net protein synthetic rates (effects on breakdown and synthesis). ANG II, cortisol and aldosterone may all reduce insulin sensitivity. Other mediators which may be implicated in human CKD and muscle function include ANG II, aldosterone and vitamin D (and the vitamin D receptor), although their precise roles in muscle protein turnover have yet to be determined. Glucocorticoids which may be increased in levels/activities within CKD may have a typical effect on muscle with an effect on strengthening insulin resistance in particular and increasing protein breakdown. The proinflammatory cytokines which antagonise normal anabolic pathways may also have a direct impact upon protein turnover in muscle in human CKD although this is difficult to evaluate. The net effect may translate to net protein losses, a reduction in nutritional status and muscle wasting. N.b. this loss of protein may come from both skeletal muscle and visceral protein tissues. The significant reduction in nutritional status is associated with increased morbidity and mortality in CKD studies. *N.B. Insulin also plays a role in protein synthesis activation within muscle (not shown in diagram,* e.g. *cross-signalling pathways with IGF-1); the adipocytokine alterations in CKD may affect insulin sensitivity and pathways involved; and androgens are implicated in muscle protein turnover and may also be reduced in CKD (*e.g. *hypogonadism in males), although not discussed in detail within this article. Further, an upregulation of myostatin, and downregulation of myogenesis, and satellite cell activities is likely.*

A number of perturbations in both traditional and novel hormonal and peptide pathways associated with metabolic-nutritional, inflammatory and cardiovascular function exist within CKD. Table 2 provides a summary of the potential and probable effects of these different factors in human CKD. Figure 2 provides a depiction of how these pathways may interact in muscle to induce protein breakdown and wasting.

**Table 2:** Table to summarise the potential and probable effects of specific mediators on kidney function, the alterations observed in human CKD; and effects on the modulation of nutritional, metabolic and haemodynamic factors and outcome

Hormonal, inflammatory or nutritional mediator	Effects of mediator on kidney function in normal state	Altered during CKD (levels/activities)	Potential or probable effects of alterations in mediators in human CKD on the following;					
			W/CX	AX	IR	HYP	DYSL	CVDR&M
Proinflammatory Cytokines	↓	↑	↑	↑	↑	↑?	↑	↑
GH-IGF-1	↑	↓	↑	?	↑	?	↑?	↑?
Angiotensin II	↑	↑	↑?	?	↑	↑	?	↑
Aldosterone	↑	↑	↑?	?	↑	↑	?	↑
Leptin	?	↑	↑?	↑	↑	↑?	↑	↑
Adiponectin	↑?	↑?	?	?	↓↑?	?	↑?	↑?
Ghrelin	?	↑↓?	↑?	↑?	?	?	?	?
Vitamin D	↑	↓	↑?	?	↑?	↑?	↑?	↑
Glucocorticoids	↑ short-term ↓chronic	↑?	↑	↑?	↑	↑	↑	↑

**Key:** W/CX = wasting/cachexia; AX = anorexia; IR = insulin resistance; HYP = hypertension; DYSL = dyslipidemia; CVDR&M = cardiovascular disease risk & mortality.

Table 2 summarises the possible and potential effects of different mediators discussed in the CKD state. In humans and *in vivo* it is difficult to establish direct and indirect causal evidence for certain effects and many are by association only. The proinflammatory cytokines appear central within many chronic disease states by supporting inflammatory processes, increasing oxidative stress and antagonising normal anabolic pathways. In progressive CKD this inflammatory response may be damaging and relate to dysfunction of different systems and pathways described. Further, as has been discussed there are many complex ‘paradoxes’ that appear to function within CKD and probably only after many long term clinical studies will these factors become clearer. For example, the levels and activities of different mediators are difficult to interpret as renal clearance maybe reduced for some peptides and/or inactive forms being synthesised. There is also the possibility of resistance locally such as in GH-IGF-1 resistance. Further, differentiating between normal, deficient and supraphysiological levels such as in the example of GH therapy in CKD requires investigation. E.g. in the healthy physiological state normal **GH-IGF-1 levels** would likely promote an increase in kidney function by maintaining normal cell growth, turnover and homeostasis. However, supraphysiological levels may have adverse effects. In CKD the GH-IGF-1 axis becomes dysregulated with relative GH resistance and a drop in IGF-1. The drop in IGF-1 can be related to both GH resistance and progressive general malnutrition in CKD, e.g. anorexia, reduced caloric intake and reduced protein intake (both therapeutically and involuntarily). **Angiotensin II, ANGII** in the healthy state has the physiological role of maintaining blood pressure and potentially other pathways such as oxidative stress and cell cycle factors. In CKD over-activation of the RAAS system may take place, which is similarly related to possible **hyperaldosteronism** in CKD. Much of the human research in CKD has been using renin angiotension system, RAS blockade studies using angiotensin-receptor blockers, ARBs and angiotensin-converting enzyme inhibitors, ACEIs., and more recently mineralcorticoid receptor, MR blockers. **Leptin** in the normal state plays a major role in “switching off” appetite and increasing energy expenditure. In CKD, hyperleptinemia and hyperactivation of anorexigenic pathways may contribute to the development of the anorexia-cachexia syndrome. Other roles are suggestible, such as effects on inflammation and potential in-direct effects on hypertension via activation of the sympathetic nervous system. In human CKD data is scarce on other direct effects. **Adiponectin** may have a beneficial effect on different pathways and insulin sensitivity. In CKD, adiponectin levels increase systemically but the relevance of this effect is unknown. Different variants of the peptide and/or adiponectin resistance may be implicated in the dysfunctional state and effects on metabolism; e.g. dysregulation of oxidative stress/inflammatory and/or insulin sensitivity factors. **Ghrelin** levels rise in CKD, however, there has been some debate in the literature as to whether the peptide is a dysfunctional variant (i.e. des-acyl ghrelin) and/or there is some level of ghrelin resistance within tissues, e.g. centrally in the hypothalamus. **Vitamin D;** studies show that prevalence of vitamin D deficiency/insufficiency is high and correlates with CVD and outcome/survival. Other new research suggests it may have multiple functions within skeletal muscle and in immune function, for example. **Glucocorticoids;** are implicated in disease pathology and stress-mediated effects; the true implications of glucocorticoid function in CKD and metabolic dysfunction has not been fully evaluated.

## Future studies in human CKD

Further research will be needed to investigate exact mechanisms and pathways of action of different mediators, their effects and interactions in human CKD from both a causative and developmental perspective of disease; and the potential for pharmacological mediation. This will include acquiring robust clinical evidence for the beneficial effects of different pharmacological agents on metabolic health and patient outcome. Suggested pharmacological agents to be evaluated may include those which favourably impact upon insulin sensitivity, CVD risk, muscle function, ACS and tissue damage/fibrosis. ANG II blockade, anabolic therapies, GH-IGF-1, GH-secretagogues and ghelin mimetics; vit D receptor agonists, insulin sensitizers such as metformin; novel therapies acting on central hypothalamic neurons, leptin and ghrelin pathways; 11β-HSD 1 inhibitors reducing corticosteroid activity pathways and MR antagonism may be of potential benefit.

The management of dyslipidemia in CKD is important as lipid abnormalities are common and may increase CVD risk and kidney pathology [14,16,140]. Current treatment includes statins and fibrates but concerns over toxicity (e.g. in muscle) [141] may initiate the combined treatment with other medications and nutrients such as omega-3 fatty acids/fish oils- especially as omega-3 fatty acids for example have potential hypo-lipidemic, anti-inflammatory, anti-thrombic, anti-atherogenic and anti-arrthymic effects [141-143].

Leading on from dyslipidemia, the effects of insulin resistance and diabetes risk in CKD must be evaluated and treatment modalities investigated, as diabetes increases risk of dyslipidemia and morbidity [141]. With regards to this the role of the different hormones and mediators described, i.e. the adipocytokines, ANG II, IGF-1, glucocorticoids, aldosterone and vit D requires investigation especially with respects to pharmacological and nutritional intervention.

Nutritional treatment should continue to be investigated as clinical outcome is clearly associated with nutritional status, and is difficult to maintain in the CKD patient group due to reduced dietary intake and abnormalities in energy, protein and mineral metabolism (due to a range of factors including many of the systems perturbed discussed here) [28,29]. A range of diagnostic criteria have been developed to assess nutritional status, presence of wasting and cachexia in CKD and these are evolving [59,60]. The malnutrition-inflammation score (MIS) was developed to assess markers of the so-called ‘malnutrition-inflammation complex syndrome’ (MICS) in ESRD dialysis patients [144]. Studies will need to clarify the effects of nutritional protein-energy supplementation and pharmacological interventions on markers of outcome using different assessment tools. Further, the nutritional complications in dialysis needs to be continually looked at as dialysis itself is catabolic and with potential for glucotoxicity due to high D-glucose composition of PD solutions [145].

The interaction of individual genetic factors, polymorphisms and levels of active mediators, and outcome requires investigation in CKD. Gene expression and activity of the proinflammatory cytokines, IL-1, -6, TNF; GH-IGF-1, the MR, GR and VDR for example would be obvious candidates for study. For example, Stenvinkel et al., 2005 showed that lower fetuin levels were associated in inflamed and malnourished ESRD patients with worsening CVD mortality outcome [146]; and that a gene polymorphism of the fetuin gene (AHSG Thr256Ser) was linked to reduced circulating fetuin in patients with higher all-cause and CVD mortality rates. Other novel markers may include the FGF-23 protein from bone which has been found to act as a marker of CKD progression [147].

Finally, the role of other therapies such as exercise (aerobic and resistance training) is yet to be fully explored and evaluated; as CKD patients tend to have reduced physical activity patterns which may potentiate the insulin resistant and cachectic state [12,13]. Physical activity may reduce morbidity and improve survival in ESRD patients [148]. Exercise is known to have potent beneficial effects on hemodynamic, endocrine (e.g. GH-IGF-1), metabolic (e.g. improve glucose disposal/control and blood lipid clearance) and skeletal muscle function and potentially reduce anemic symptoms and inflammation [12,65-67,134] and hence should be seriously considered as a form of therapy in CKD in conjunction with pharmacology and nutrition.

## Conclusion

CKD is associated with high CVD risk and overall morbidity and mortality. A number of hormonal, inflammatory and nutritional factors and mediators have been implicated in the development of metabolic and hemodynamic dysfunction (e.g. insulin resistance, dyslipidemia and hypertension), malnutrition, anorexia-cachexia, CVD risk and patient outcome. These may include chronic inflammation and high proinflammatory cytokine production (and levels of IL-1β, IL-6 and TNF-α), altered hepatic acute phase protein levels such as reduced albumin and increased CRP; hyperactivation of the RAAS; disturbances of normal function and activities of the GH-IGF-1 axis (GH-IGF-1 resistance), the adipocytokines leptin and adiponectin, the gut-derived appetite hormone ghrelin; reduced vit D status/activities and increased glucocorticoid activity (and MR activity) may also be involved. In addition, clearly nutritional factors including protein (such as reduced protein intake) and amino acids (perturbed amino acid metabolism) are altered but have not been discussed in detail within this review. There may be real possibilities emerging for reducing risk of CKD development by focusing on reducing obesity and MetS prevalence and pharmacologically focusing on specific systems discussed within this review and their interactions with other therapies such as nutrition and exercise.

## Abbreviations

APRs: Acute phase reactants; ACTH: Adrenocorticotropin hormone; AMPK: AMP-activated protein kinase; ACEI: Angiotensin converting enzyme inhibitors; ANG II: Angiotensin II; ARB: Angiotensin II receptor blocker; ATII-1 and −2 R: Angiotensin II receptors type 1 and 2; ACS: Anorexia-cachexia syndrome; 11β-HSD: 11βeta-hydroxysteroid dehydrogenase; BMI: Body mass index; CVD: Cardiovascular disease; CHF: Chronic heart failure; CKD: Chronic kidney disease; CBG: Corticosteroid binding globulin; CRH: Corticotrophin-releasing hormone; CRP: C-reactive protein; EGFR: Estimated glomerular filtration rate; ESRD: End-stage renal disease; EPO: Erythropoietin; GH: Growth hormone; REE: Resting energy expenditure; HRV: Heart rate variability; IGF-I: Insulin-like growth factor-I; IFNγ: Interferon gamma; IL-1β: Interleukin – 1beta; IL-6: Interleukin −6; LBM: Lean body mass; MBD: Mineral bone disease; MR: Mineralcorticoid receptor; PPARγ: Peroxisome proliferator-activated receptor γ; ROS: Reactive oxygen species; RAAS: Renin angiotensin aldosterone system; S-Alb: Serum albumin; TZDs: Thiazolidinediones; TNF-α: Tumor necrosis factor – alpha; VDR: Vitamin D receptor.

## Competing interests

The author declares that there are no competing interests.

## Author contribution

Dr Adrian D Slee is the sole author and contributor.
